# Epstein–Barr Virus and Human Cytomegalovirus Infection in Intestinal Mucosa of Chinese Patients With Inflammatory Bowel Disease

**DOI:** 10.3389/fmicb.2022.915453

**Published:** 2022-05-31

**Authors:** Wei Wang, Xin Chen, Jie Pan, Xianhui Zhang, Liyun Zhang

**Affiliations:** ^1^Department of Laboratory Medicine, Shanxi Provincial People’s Hospital, Taiyuan, China; ^2^Department of Laboratory Medicine, The 908th Hospital of Chinese PLA Joint Logistics Support Force, Nanchang, China; ^3^Department of Pathology, Stanford University School of Medicine, Palo Alto, CA, United States; ^4^Department of Rheumatology, Third Hospital of Shanxi Medical University, Shanxi Bethune Hospital, Shanxi Academy of Medical Sciences, Tongji Shanxi Hospital, Tongji Medical College, Huazhong University of Science and Technology, Taiyuan, China

**Keywords:** Epstein–Barr virus, cytomegalovirus, inflammatory bowel disease, disease activities, viral load

## Abstract

**Objective:**

This study aimed to determine the frequency of Epstein–Barr virus (EBV), cytomegalovirus (CMV) in mucosa and blood of inflammatory bowel disease (IBD) patients in China and evaluate their correlation with the clinical disease activities.

**Methods:**

Peripheral blood and endoscopic fresh colonic mucosal samples were collected from a cohort of 287 IBD patients and 50 controls. Viral DNA load was analyzed through quantitative real-time PCR. The clinical disease activity of ulcerative colitis (UC) and Crohn’s disease (CD) was assessed by the Mayo Clinic Score and Crohn’s disease activity index, respectively.

**Results:**

Among 287 IBD patients, 228 (79.4%) were positive for EBV and 99 (34.5%) were positive for CMV. EBV and CMV infection rates are significantly higher than those in the control group (28.0%, *p* < 0.05; 4.0%, *p* < 0.05). In addition, EBV/CMV prevalence increases as clinical activities progress [For EBV infection, the prevalence was 53.93% (48/89) in the mild group, 87.00% (87/100) in the moderate group, and 94.90% (93/98) in the severe group; and for CMV infection, the prevalence was 3.37% (3/89) in the mild group, 27.00% (27/100) in the moderate group, and 70.41% (69/98) in the severe group]. EBV and CMV loads are related to clinical disease activities (*p* < 0.05). In addition, viral load in the intestinal mucosa of patients with acute exacerbation of IBD is higher than that of patients in remission.

**Conclusion:**

High prevalence of EBV and CMV is found in patients with IBD, and their prevalence is related to clinical disease activities. In addition, the viral load in the intestinal mucosa is associated with the status of mucosa in the same patients (active phase versus remission phase). Detection of viral load on mucosal specimens with quantitative real-time PCR is a feasible method to monitor EBV and CMV infection in IBD patients.

## Introduction

Inflammatory bowel disease (IBD), namely, ulcerative colitis (UC) and Crohn’s disease (CD), is a common disease in North America, Oceania, and many European countries. In recent years, the incidence of IBD has also been rising in developing countries in Asia, South America, and Africa (Kaplan, 2015; Ng et al., 2017; Windsor and Kaplan, 2019; Hodges and Kelly, 2020). Several concerns commonly associated with IBD, including repeated hospitalizations, drug effects, surgery, stoma formation, and fear of being a burden, can have many adverse effects on the quality of life of patients with IBD (Mohsenizadeh et al., 2020). Therefore, patients with IBD need long-term, systematic treatment. The pathogenesis of IBD has not been fully elucidated. It is currently believed that IBD is an emerging chronic and disabling enteropathy triggered and sustained by the interaction of environmental, genetic, infectious, and immune factors (Chang, 2020).

In contrast to the evidence for the role of gut microbiota in IBD pathogenesis, the involvement of the gut virome is only beginning to be investigated (Lopetuso et al., 2016; Ungaro et al., 2019). The infections of Epstein–Barr virus (EBV) and human cytomegalovirus (CMV) are common and usually acquired early in life. Therefore, the status of EBV and CMV infections in the intestinal mucosa of patients with IBD and its role in disease progression has attracted extensive attention. The reported frequency of CMV infection ranged from 21% to 34% in severe acute UC and 33%–36% in refractory cases (Kandiel and Lashner, 2006; Kim et al., 2010), while EBV was higher, ranging from 33% to 81% (Ryan et al., 2012; Li et al., 2018). Despite some association between CMV or EBV infection and severe colitis, the relationship between the virus and IBD is less well-defined. The role of these viruses in IBD, either as contributors or innocent bystanders, remains a topic of ongoing controversy. The clinical characteristics, risk factors, and treatment outcomes of IBD patients complicated with EBV and CMV infection remain to be explored.

So far, the data on the positive rates of CMV and EBV in patients with IBD vary considerably due to different study designs or detection methods. In particular, there are few studies on the viral infection rate in the intestinal mucosa of IBD patients in China. Therefore, this study aimed to investigate CMV and EBV infection status in the intestinal mucosa of Chinese IBD patients and discuss the risk factors and significance of antiviral therapy for IBD with CMV and EBV infection.

## Materials and Methods

### Patient Tissue and Blood Sample

From May 2016 to September 2021, a cohort of 287 IBD patients (aged 14–75 with a mean of 46.5 ± 15.2 years) and 50 controls (aged 18–69 with a mean of 48.8 ± 12.5 years) underwent peripheral blood and endoscopic fresh colonic mucosal sample harvest at the Shanxi Provincial People’s Hospital. Patient inclusion criteria were based on widely accepted criteria for the IBD diagnosis, including their clinical, endoscopic, and histological features (Gomollón et al., 2017; Magro et al., 2017; Wu et al., 2018). In addition, matched subjects who underwent colonoscopy for screening for polyps or irritable bowel syndrome were recruited as controls. The study was approved by the ethical committee of the Shanxi Provincial People’s Hospital. The severity of patients with ulcerative colitis (UC) was assessed using the Mayo Clinic score (D’Haens et al., 2007), and the patients with CD were assessed using the Crohn’s disease activity index (Best et al., 1976).

### Quantitative Real-Time PCR as a Measure of CMV and EBV Viral Load

The quantitative determination of EBV DNA and CMV DNA was performed by real-time PCR on peripheral blood and fresh colonic mucosal samples using the EBV PCR Fluorescence Quantitative Diagnostic Kit (Sansure Biotech Inc., Hunan, China) and CMV PCR Fluorescence Quantitative Diagnostic Kit (Sansure Biotech Inc., Hunan, China). qRT-PCR was performed using Bio-Rad CFX-96 Real-Time PCR instruments with 50 μl reaction volume containing 37 μl reaction solution, 2 μl enzyme mix, 1 μl internal standard, and 10 μl DNA extraction. PCR reactions were performed using the following conditions: 50°C for 2 min, 94°C for 5 min, followed by 45 cycles at 94°C for 15 s and 57°C for 30 s. The results of peripheral blood were shown as viral DNA copies/ml, and results of tissue were shown as viral DNA copies/mg tissue.

### Statistical Analyses

Categorical variables were presented as counts and percentages, and continuous variables were described as median and interquartile ranges. The prevalence of EBV or CMV was analyzed by the Binary Logistic Regression method and chi-squared analysis. The viral load was log10 transformed before analysis, and the differences among groups were analyzed by Kruskal–Wallis H-test and Mann–Whitney U-test, as appropriate. The SPSS software version 22.0 (IBM Company, Chicago, IL, United States) was used to analyze all data, and the two-sided value of *p* less than 0.05 was considered statistically significant.

## Results

### General Information and EBV/CMV Mucosal Infection in IBD Patients

A total of 287 IBD patients and 50 control subjects are presented in Table 1 were recruited. Among IBD patients, 192 were diagnosed with UC and 95 had CD. Compared with IBD patients (46.5 ± 15.2 years), the mean age of the control group was 48.8 ± 12.5 years old. The mean illness duration of IBD patients was 46.5 ± 15.2 months.

**Table 1:** Characteristics of inflammatory bowel disease (IBD) patients.

	Total	Types of IBD^a^		Controls
		UC^a^	CD^a^	
Number of patients	287	192	95	50
Male/female	171/116	111/81	60/35	32/18
Age (years)	46.5 ± 15.2	48.9 ± 14.8	41.5 ± 14.9	48.8 ± 12.5
Illness duration (month)	30 (13–100)	28 (12–100)	50 (13–103)	NA
EBV +	228 (79.4%)	157 (81.8%)	71 (74.7%)	14 (28.0%)
CMV +	99 (34.5%)	86 (44.8%)	13 (13.7%)	2 (4.0%)

a IBD, inflammatory bowel disease; UC, ulcerative colitis; and CD, Crohn’s disease.

With real-time PCR, we observed the prevalence of EBV and CMV in the colonic mucosa of IBD patients. Among 287 IBD patients, 228 (79.4%) were positive for EBV, and 99 (34.5%) were positive for CMV. The infection rates of EBV and CMV are significantly higher than those in the control group (28.0%, *p* < 0.05; 4.0%, *p* < 0.05). EBV positivity in colonic mucosa was 81.8% (157/192) in UC and 74.7% (71/95) in CD, and CMV positivity in colonic mucosa was 44.8% (86/192) in UC and 13.7% (13/95) in CD (Table 1).

### Clinical Features of IBD Patients Infected With EBV and CMV

Among 197 UC patients, 32 were negative for both EBV and CMV, 74 were singly infected with EBV, and 83 were positive for both EBV and CMV (patients positive for CMV and negative for EBV were not counted due to small sample size). First, we analyzed the differences in Clinical features among groups. There were significant differences in clinical disease activities, hemoglobin, erythrocyte sedimentation rate, C-reaction protein, albumin, and serum potassium among the three groups (*p* < 0.05). At the same time, there were no significant differences in gender, age, illness duration, location, clinical classification, and white blood cell among the three groups (*p* > 0.05). In addition, the data showed that hemoglobin, albumin and serum potassium were higher, and the erythrocyte sedimentation rate and C-reaction protein were lower for patients with EBV infection compared to the patients with EBV and CMV infection (*Z* = −4.367, *p* < 0.05; *Z* = −2.455, *p* < 0.05; *Z* = −4.207, *p* < 0.05; *Z* = −3.858, *p* < 0.05); while compared with those uninfected patients, albumin was lower and the erythrocyte sedimentation rate and C-reaction protein were higher in patients with EBV infection (*Z* = −2.357, *p* < 0.05; *Z* = −2.285, *p* < 0.05; *Z* = −2.935, *p* < 0.05; Table 2).

**Table 2:** Clinical features of UC^a^ patients infected with Epstein–Barr virus (EBV) and cytomegalovirus (CMV).

	Total (*n* = 192)	EBV−CMV− (*n* = 32)	EBV + CMV− (*n* = 74)	CMV + EBV− (*n* = 3)	EBV + CMV+ (*n* = 83)	*p* *^c^*
Male/female	111/81	15/17	40/34	3/0	53/30	0.14
Age (years)	48.9 ± 14.8	47.7 ± 18.1	50.8 ± 13.4	49.0 ± 20.5	47.8 ± 14.5	0.61
Illness duration (months)	28 (12–100)	20 (10–100)	30 (16–113)	90 (18-)	21 (10–82)	0.12
Location^b^						0.07
E1^b^	14 (7.29%)	4 (12.50%)	5 (6.76%)	1 (33.33%)	4 (4.82%)	
E2^b^	31 (16.15%)	6 (18.75%)	6 (8.11%)	0	19 (22.89%)	
E3^b^	147 (76.56%)	22 (68.75%)	63 (85.14%)	2 (66.67%)	60 (72.29%)	
Clinical disease activities						0.00
Mild	34 (17.71%)	19 (59.38%)	13 (17.57%)	0	2 (2.41%)	
Moderate	70 (36.46%)	10 (31.25%)	37 (50.00%)	1 (33.33%)	22 (26.51%)	
Severe	88 (45.83%)	3 (9.38%)	24 (32.43%)	2 (66.67%)	59 (67.47%)	
Clinical classification						0.71
Incipient	28 (14.58%)	6 (18.75%)	9 (12.16%)	0	13 (15.66%)	
Chronic	164 (85.42%)	26 (81.25%)	65 (87.84%)	3 (100%)	70 (84.34%)	
*laboratory examination*						
White blood cell (×10^9^/L)	6.68 (5.38–8.71)	6.22 (5.07–7.91)	6.26 (5.05–8.59)	7.00 (6.55-)	7.01 (5.98–9.32)	0.13
Hemoglobin (g/L)	125 (106–140)	134 (124–143)	130 (110–144)	138 (122-)	114 (96–132)	0.00
Erythrocyte sedimentation rate (mm/h)	18 (10–30)	9 (5–16)	15 (8–24)	22 (2-)	24 (16–40)	0.00
C-Reaction protein (mg/dL)	6.56 (2.68–20.14)	2.49 (0.76–3.82)	5.03 (1.59–16.99)	3.50 (0.55-)	15.56 (5.25–44.90)	0.00
Albumin (g/L)	36.27 (30.66–40.72)	39.55 (38.11–42.40)	37.55 (34.15–41.13)	33.07 (22.12-)	32.56 (27.58–37.70)	0.00
Serum potassium (mmol/L)	3.68 (3.37–4.00)	3.84 (3.52–4.00)	3.73 (3.50–4.05)	3.12 (2.62-)	3.64 (3.22–3.87)	0.01

a UC, ulcerative colitis.

b E1, ulcerative proctitis; E2, left-sided UC; and E3, extensive UC.

c Patients positive for CMV and negative for EBV was not counted due to small sample size.

Among 122 CD patients, 24 were negative for both EBV and CMV, 58 were singly infected with EBV, and 13 were positive for both EBV and CMV. There were significant differences in illness duration, clinical disease activities, hemoglobin, and albumin among the three groups (*p* < 0.05). At the same time, there were no significant differences in gender, age, location, clinical classification, white blood cell, erythrocyte sedimentation rate, C-reaction protein, and serum potassium among the three groups (*p* > 0.05). Hemoglobin and albumin were higher for patients with EBV infection than patients with EBV and CMV infection (*Z* = −2.566, *p* < 0.05; *Z* = −2.527, *p* < 0.05). There was no statistical difference between patients with EBV infection and uninfected patients in the above indicators (*p* > 0.05; Table 3).

**Table 3:** Clinical features of CD^a^ patients infected with EBV and CMV.

	Total (*n* = 95)	EBV−CMV− (*n* = 24)	EBV + CMV− (*n* = 58)	EB + CMV+ (*n* = 13)	*p*
Male/female	60/35	16/8	36/22	8/5	0.92
Age (years)	41.5 ± 14.9	40.88 ± 16.63	41.55 ± 13.83	42.08 ± 17.02	0.97
Illness duration (months)	50 (13–103)	78 (34–144)	50 (17–101)	10 (6–22)	0.00
Location^b^					0.07
L1^b^	40 (42.11%)	13 (54.17%)	23 (39.66%)	4 (30.77%)	
L2^b^	18 (18.95%)	3 (12.50%)	9 (15.52%)	6 (46.15%)	
L3^b^	37 (38.95%)	8 (33.33%)	26 (44.83%)	3 (23.08%)	
L4^b^	0	0	0	0	
Clinical disease activities					0.00
Mild	55 (57.89%)	22 (91.67%)	32 (55.17%)	1 (7.69%)	
Moderate	30 (31.58%)	2 (8.33%)	24 (41.38%)	4 (30.77%)	
Severe	10 (10.53%)	0 (%)	2 (3.45%)	8 (61.54%)	
Clinical classification					0.34
Incipient	16 (16.84%)	4 (16.67%)	8 (13.79%)	4 (30.77%)	
Chronic	79 (83.16%)	20 (83.33%)	50 (86.21%)	9 (69.23%)	
*Laboratory examination*					
White blood cell (×10^9^/L)	6.10 (5.07–7.96)	5.65 (4.36–6.60)	6.56 (5.09–8.47)	5.60 (5.28–7.82)	0.07
Hemoglobin (g/L)	126 (113–140)	132 (117–140)	129 (114–144)	108 (89–125)	0.03
Erythrocyte sedimentation rate (mm/h)	14 (6–32)	11 (5–25)	14 (7–33)	30 (11–56)	0.12
C-Reaction protein (mg/dL)	4.80 (1.01–17.50)	3.34 (0.93–78.57)	5.12 (0.87–33.80)	15.20 (4.02–103.70)	0.08
Albumin (g/L)	40.00 (34.90–43.56)	40.25 (38.16–44.00)	40.39 (35.34–43.53)	29.76 (26.71–41.03)	0.03
Serum potassium (mmol/L)	3.86 (3.58–4.17)	3.93 (3.59–4.27)	3.88 (3.61–4.11)	3.58 (3.25–4.08)	0.17

a CD, Crohn’s disease.

b L1, terminal ileum; L2, colon; L3, Ileo-colon; and L4, upper gastrointestinal location.

### EBV and CMV Prevalence and Viral Load Among Groups According to the Clinical Disease Activities

**Figure 1:**
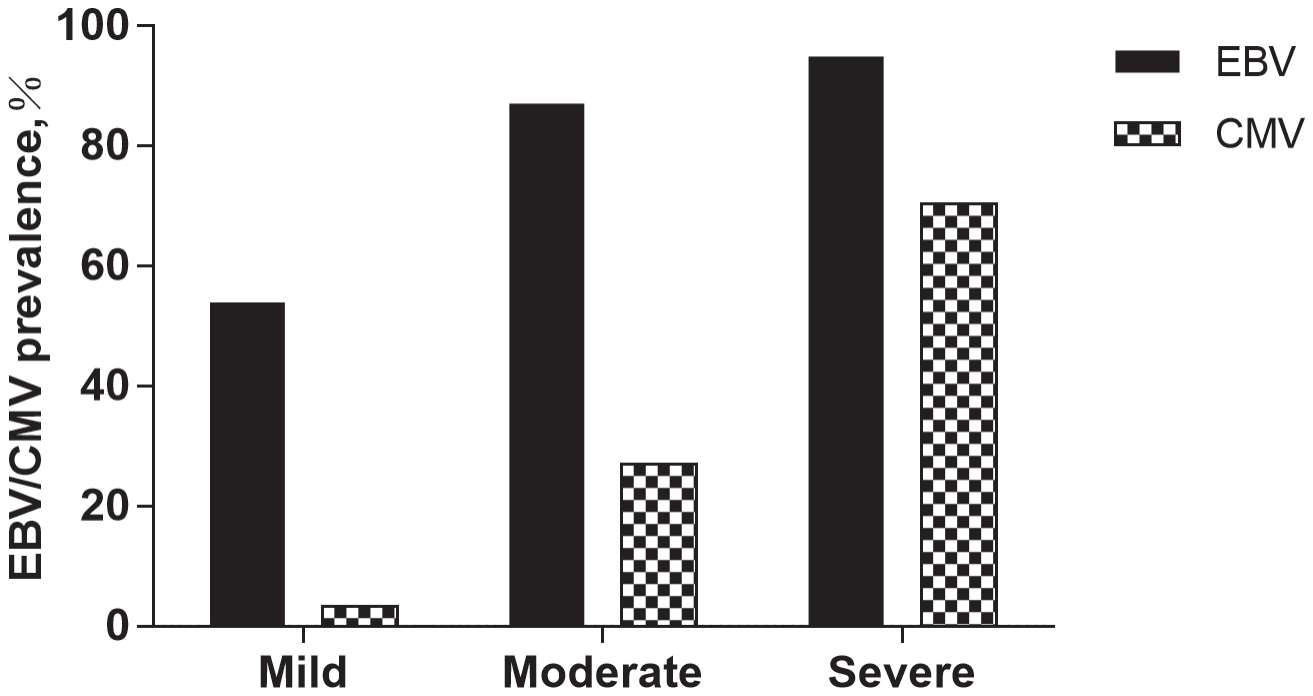
EBV/CMV prevalence among groups according to the clinical disease activities. The prevalence of EBV is 53.93% (48/89) in the mild group, 87.00% (87/100) in the moderate group, and 94.90% (93/98) in the severe group. The prevalence of CMV is 3.37% (3/89) in the mild group, 27.00% (27/100) in the moderate group, and 70.41% (69/98) in the severe group. EBV/CMV prevalence increases as clinical activities progress (*p* < 0.05).

We analyzed the relationships between viral prevalence or viral load (log10 transformed) and the patients’ degree of clinical disease activities (Figure 1). Irrespective of the type of IBD, the prevalence of EBV is 53.93% (48/89) in the mild group, 87.00% (87/100) in the moderate group, and 94.90% (93/98) in the severe group. The prevalence of CMV is 3.37% (3/89) in the mild group, 27.00% (27/100) in the moderate group, and 70.41% (69/98) in the severe group. EBV/CMV prevalence increases as clinical activities progress (*p* < 0.05).

**Figure 2:**
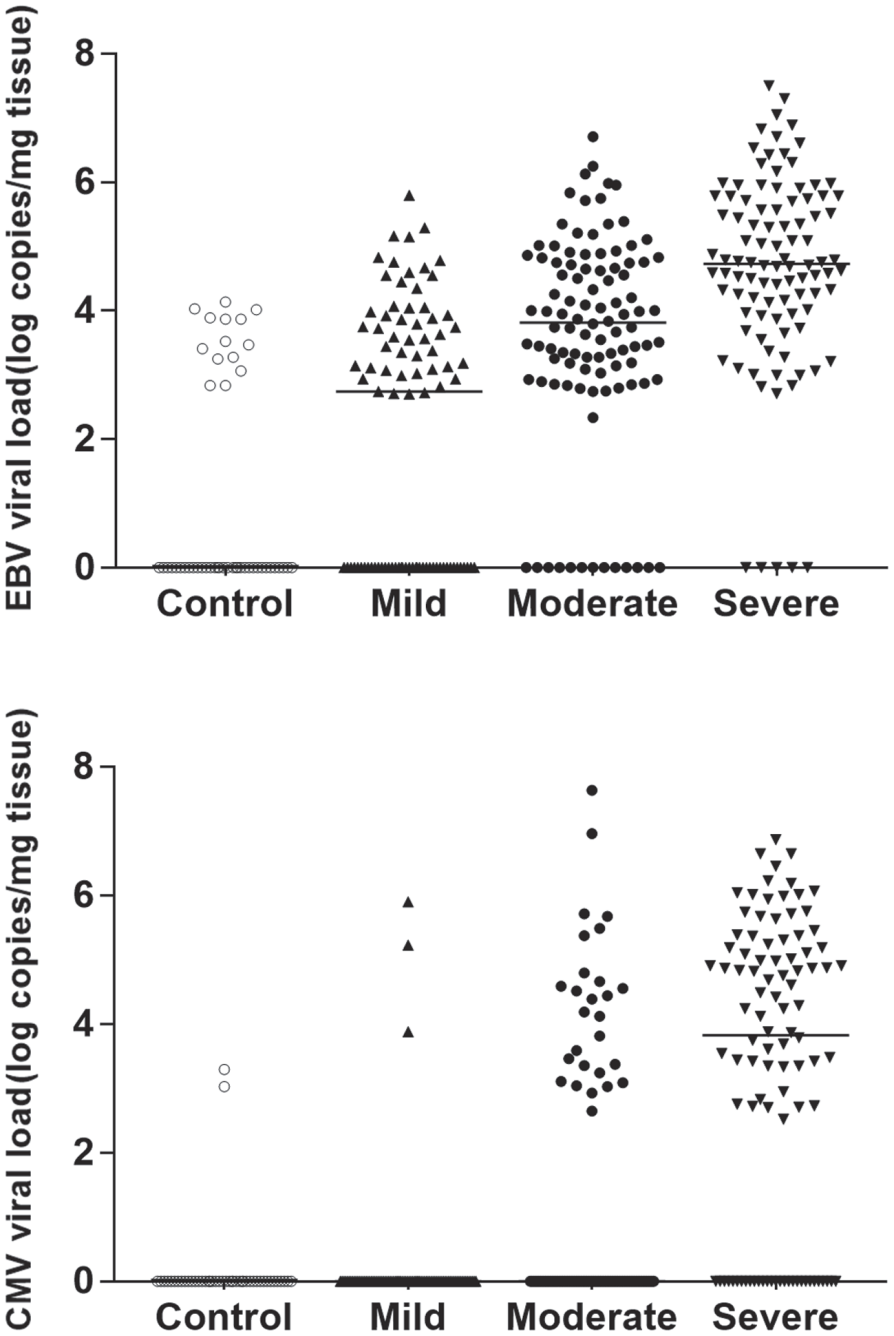
EBV/CMV viral load among groups according to the clinical disease activities. The EBV viral loads of the control group, mild group, moderate group, and severe group were 0.00 (0.00–2.90), 2.74 (0.00–3.77), 3.82 (2.96–4.76), and 4.73 (3.97–5.75), respectively. The CMV viral loads of the control group, mild group, moderate group, and severe group were 0.00 (0.00–0.00), 0.00 (0.00–0.00), 0.00 (0.00–3.01), and 3.83 (0.00–5.19), respectively. EBV and CMV loads are related to clinical disease activities (*p* < 0.05).

With the real-time, we also analyzed the viral loads of EBV and CMV in the colonic mucosa of IBD patients (Figure 2). The EBV viral loads of the control group, mild group, moderate group, and severe group were 0.00 (0.00–2.90), 2.74 (0.00–3.77), 3.82 (2.96–4.76), and 4.73 (3.97–5.75), respectively. The CMV viral loads of the control group, mild group, moderate group, and severe group were 0.00 (0.00–0.00), 0.00 (0.00–0.00), 0.00 (0.00–3.01), and 3.83 (0.00–5.19), respectively. EBV and CMV loads are related to clinical disease activities (*p* < 0.05).

### EBV and CMV Viral Loads of IBD Patients in the Active Phase and Remission Phase

**Figure 3:**
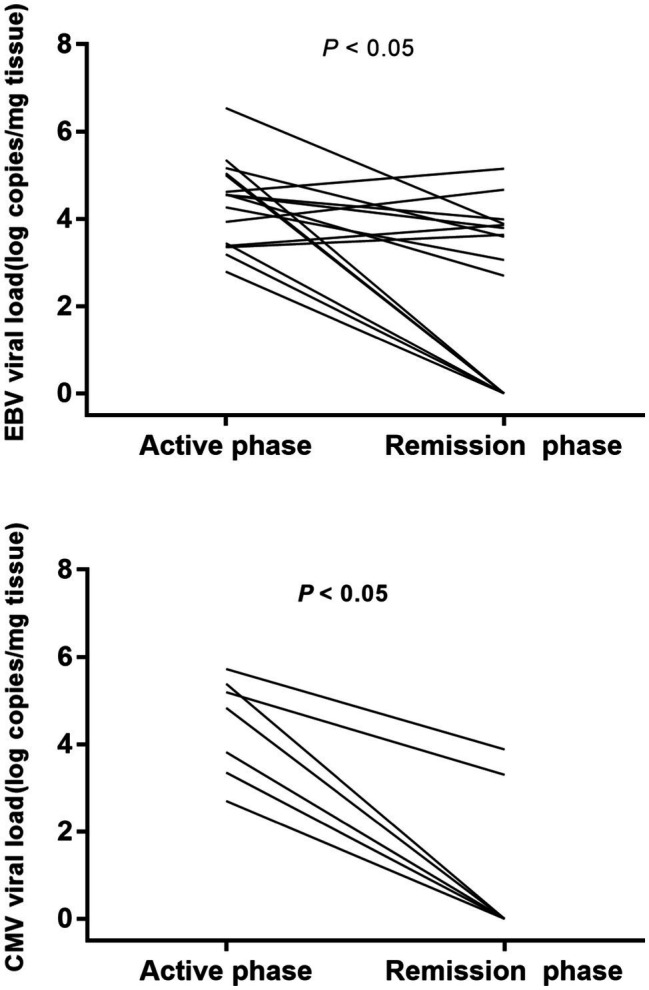
EBV and CMV viral load of IBD patients in the active and remission phases. Viral loads decreased in most patients with EBV colitis and all patients with CMV colitis.

We observed EBV and CMV viral loads of 16 IBD patients in the active and remission phases (Figure 3). All patients (16 positives for EBV and 7 for CMV) achieve remission (Mayo Clinic score ≤2 for UC, Crohn’s disease activity index<150 for CD) on comprehensive therapy, including ganciclovir antiviral therapy. The viral loads were reduced in most patients with EBV colitis (six cases turned negative and six cases had reduced viral load); meanwhile, the viral loads have also shown a dramatic decline in all patients with CMV colitis (five cases turned negative and two cases had reduced viral load).

### Peripheral Blood Viral Detection

The prevalence of EBV and CMV in peripheral blood of IBD patients was also detected. The EBV and CMV viral loads in peripheral blood were 3.06 (0.00–3.83) and 0.00 (0.00–0.00) respectively. Among 287 IBD patients, 150 were positive for EBV and 20 were positive for CMV. The prevalence of EBV is 19.10% (17/89) in the mild group, 56.00% (56/100) in the moderate group, and 78.57% (77/98) in the severe group. The prevalence of CMV is 0.00% (0/89) in the mild group, 5.00% (5/100) in the moderate group, and 15.31% (15/98) in the severe group. All these patients with positive virus in peripheral blood carried the same virus at the level of colonic mucosa. The sensitivity of real-time PCR on peripheral blood samples to detect CMV and EBV compared to that on mucosa tissue samples was 20.2% and 65.8%, respectively, and the specificity was 100%.

### Risk Factors for EBV and CMV Infection in the Colonic Mucosa of IBD Patient

The potential risk factors for viral infection in IBD patients were assessed. Clinical disease activity was identified as a significant risk factor for both EBV and CMV colitis (*p* < 0.05, OR = 4.090 and 5.638, 95% CI: 2.364–7.070 and 3.405–9.333, respectively). No significant associations were found between an increased risk of viral infection and the gender, age, hemoglobin, albumin, disease duration, steroid therapy, biologic agent therapy, and immunosuppressive therapy (Table 4).

**Table 4:** Risk factors for EBV and CMV infection in the colonic mucosa of IBD^a^ patient.

Variable	EBV infection			CMV infection		
	OR	95% CI	*p*	OR	95% CI	*p*
Gender	0.833	0.398–1.745	0.63	0.829	0.415–1.655	0.60
Age	1.006	0.984–1.029	0.59	1.005	0.984–1.027	0.63
Hemoglobin	0.987	0.967–1.008	0.22	0.994	0.977–1.012	0.52
Albumin	1.002	0.937–1.072	0.95	0.935	0.881–0.992	0.03
Illness duration	0.999	0.996–1.001	0.28	0.999	0.997–1.001	0.41
Clinical disease activities	4.090	2.364–7.077	0.00	5.638	3.405–9.333	0.00
Steroid therapy	1.091	0.428–2.782	0.86	1.184	0.592–2.371	0.63
Biologic agent therapy	0.711	0.325–1.555	0.39	1.336	0.582–3.065	0.49
Immunosuppressant therapy	0.667	0.189–2.352	0.53	1.045	0.265–4.129	0.95

a IBD, inflammatory bowel disease.

## Discussion

EBV and CMV, which are members of the human herpesvirus family, are prevalent in healthy individuals. However, in recent years, despite EBV and CMV infection have attracted more and more attention in IBD, several key issues remain unresolved, such as their prevalence and role in IBD, optimal diagnosis and treatment methods, and the possible risk factors. To date, few kinds of research have explored EBV and CMV colonic infection in Chinese IBD patients.

The reported frequency of CMV infection ranged from 21% to 36%, and EBV ranged from 33% to 81% (Kandiel and Lashner, 2006; Kim et al., 2010; Ryan et al., 2012; Li et al., 2018). Our study shows that 79.4% of patients have EBV infection, and 34.5% have CMV infection in the colonic mucosa of IBD patients. The differences in infection rates among different studies may be related to the different patient profiles and the diagnostic methods used in the studies. In the earlier studies, immunohistochemistry was the most commonly used method (Vega et al., 1999; Yanai et al., 1999; Hazr-Konya et al., 2021). However, it has recently been demonstrated that quantitative real-time PCR analysis of nucleic acids extracted from fresh colonic mucosal samples can improve the sensitivity, specificity, and repeatability. With real-time PCR, our study showed that the infection rates of EBV and CMV in 192 patients with UC were 81.8% and 44.8%, and the infection rates in 95 patients with CD were 44.8% and 13.7%. These data suggest that the infection rate of UC patients may be higher than that of CD patients, both of which are higher than the control group. From an anatomical point of view, in patients with UC, the damage to the rectum is constant and the lesions to the colon are variable and continuous, while in patients with CD, the lesions of the whole digestive tract are discontinuous (Jentzer et al., 2020). Immunologically, in patients with CD, IFN-γ produced by a T helper cell Th1/Th17 shows antiviral properties, while in patients with UC, the Th2/Th9 profile does not prevent viral replication (Fuss, 2011; Rossini et al., 2012). These differences may lead to higher viral infection rates in UC than in CD.

The clinical manifestations of IBD patients are one of the bases for diagnosing EBV and CMV virus infection. Our study reveals a correlation between clinical disease activities and EBV/CMV infection in the colonic mucosa of IBD patients. The data showed that IBD patients with CMV and EBV infection had lower hemoglobin levels, serum albumin, and serum potassium, and higher levels of erythrocyte sedimentation rate and C-reactive protein, suggesting that patients with EBV and CMV infection may have more severe clinical manifestations and inflammatory reactions. In IBD patients, mucosal damage may impair intestinal mechanical barrier function, leading to viral infection. In addition, the role of T cells in controlling viral replication may be compromised by immunosuppressive drugs and high clinical disease activity, resulting in high viral infection rates (Vogl et al., 2011; Lopetuso et al., 2016).

Our study also reveals that the prevalence of EBV and CMV increased with the progress of clinical disease activities of IBD. The prevalence of EBV is 53.93%, 87.00%, and 94.90% in the mild group, moderate group, and severe group, and the prevalence of CMV is 3.37%, 27.00%, and 70.41% in the three groups. According to clinical disease activity, further quantitative analysis of the viral load in different groups revealed that the severe group had a higher viral load. Therefore, active viral replication may exacerbate the histopathological changes and intestinal mucosal lesions in IBD. In patients with IBD, the EBV virus can infect both intestinal epithelial cells and B cells, where productive viral replication is associated with direct cytopathogenic effects (Ciccocioppo et al., 2015b). Thus, in severe patients, EBV replication in the intestinal tract is associated with the infiltration and proliferation of B cells in the inflammatory colonic mucosa (Sankaran-Walters et al., 2011; Temple et al., 2014). As for CMV infection, the virus appears to be involved in worsening IBD, but its role is unclear. The development of a vicious circle between CMV infection and inflammation has been reported, suggesting that CMV infection may influence the evolution of IBD by triggering acute flare-ups (Vega et al., 1999; Söderberg-Nauclér, 2014; Jentzer et al., 2020).

We observed EBV and CMV viral loads of 16 IBD patients who achieved remission on comprehensive therapy, including ganciclovir antiviral therapy. The data show that the viral load in the intestinal mucosa of patients with acute exacerbation of IBD is higher than that of patients in remission, suggesting the virus may have a potential role in exacerbating the disease (Ciccocioppo et al., 2015a,b). Our further analysis found that the CMV load reduction appeared to be more dramatic in patients in remission than the EBV load (Viral loads decreased in all patients with CMV colitis and most patients with EBV colitis). This may be related to the fact that the EBV virus is more involved in the onset of disease than in the clinical evolution of IBD (Lopes et al., 2017).

Considering the detection of viruses in colonic mucosa is invasive, the virus in peripheral blood of IBD patients was also detected. However, the sensitivity of peripheral blood tests is too low to meet the needs of clinical surveillance of viruses (20.2% for CMV and 65.8% for EBV). Moreover, as the report by Hong et al., about 22.2% of the hospitals in China cannot possess testing facilities for histology (Yang and Qian, 2022). Therefore, non-invasive detection with high sensitivity is needed in clinical diagnosis and treatment. In future studies, we will explore the detection of DNA viral load in stool samples to avoid invasive endoscopic detection.

Limited by the cross-sectional design of this study, most data on the prevalence of virus infection were collected simultaneously, making it unsuitable for observing the dynamic nature of infection. In addition, since a positive PCR result did not necessarily indicate an active EBV or CMV replication, whether a threshold needs to be established to confirm that the patient was suffering from an active EBV or CMV end-organ disease also needs to be discussed. The role of EBV and CMV in IBD, either as contributors or innocent bystanders, remains to be explored. Further longitudinal and molecular mechanism studies are needed. In addition, this single-center study partially reflects EBV and CMV infections among IBD patients in provincial cities in China, where the percentages of patients with moderate IBD are significantly higher than in the prefectural-level city, which may result in selection bias (Yang and Qian, 2022).

In summary, our data show that a high prevalence of EBV and CMV is found in patients with IBD and that their prevalence is related to clinical disease activities. In addition, the viral load in the intestinal mucosa is also associated with the status of mucosa in the same patients (active phase versus remission phase). Detection of viral load on freshly harvested mucosal specimens with quantitative real-time PCR is a feasible method to monitor EBV and CMV infection in IBD patients. Other non-invasive assays need to be further explored.

## Data Availability Statement

The original contributions presented in the study are included in the article/supplementary material, further inquiries can be directed to the corresponding author.

## Ethics Statement

The studies involving human participants were reviewed and approved by the Ethical Committee of Shanxi Provincial People’s Hospital. Written informed consent for participation was not required for this study in accordance with the national legislation and the institutional requirements.

## Author Contributions

WW, XC, and XZ contributed to literature search, conception and design, experiment, analysis, and the initial draft of the manuscript. JP contributed to statistical analysis. LZ contributed to edited drafts of the manuscript. All authors contributed to the article and approved the submitted version.

## Conflict of Interest

The authors declare that the research was conducted in the absence of any commercial or financial relationships that could be construed as a potential conflict of interest.

## Publisher’s Note

All claims expressed in this article are solely those of the authors and do not necessarily represent those of their affiliated organizations, or those of the publisher, the editors and the reviewers. Any product that may be evaluated in this article, or claim that may be made by its manufacturer, is not guaranteed or endorsed by the publisher.
